# Habitual dietary lactose and galactose intakes in association with age at menopause in non-galactosemic women

**DOI:** 10.1371/journal.pone.0214067

**Published:** 2019-03-19

**Authors:** Marzieh Rostami Dovom, Nazanin Moslehi, Parvin Mirmiran, Fereidoun Azizi, Fahimeh Ramezani Tehrani

**Affiliations:** 1 Reproductive Endocrinology Research Center, Research Institute for Endocrine Sciences, Shahid Beheshti University of Medical Sciences, Tehran, Iran; 2 Nutrition and Endocrine Research Center, Research Institute for Endocrine Sciences, Shahid Beheshti University of Medical Sciences, Tehran, Iran; 3 Department of Clinical Nutrition and Dietetics, Faculty of Nutrition Sciences and Food Technology, National Nutrition and Food Technology Research Institute, Shahid Beheshti University of Medical Sciences, Tehran, Iran; 4 Endocrine Research Center, Research Institute for Endocrine Sciences, Shahid Beheshti University of Medical Sciences, Tehran, Iran; Yeungnam University, REPUBLIC OF KOREA

## Abstract

**Introduction:**

Rodent models and studies on women with galactosemia suggest the ovo-toxicity effect of galactose. However, the association between galactose intake from dietary sources and the ovarian function in women without galactosemia has not yet been described. Therefore, this study aimed to investigate the associations between both dietary galactose and lactose intake, and ovarian dysfunction as the odds of early menopause in women without galactosemia.

**Materials and methods:**

This cross-sectional study was conducted on 821 women without galactosemia, participants of the Tehran Lipid and Glucose Study (TLGS), who experienced natural menopause. Habitual dietary intakes of lactose and galactose during the past 12 month were assessed, using a food-frequency questionnaire (FFQ). In this study, early menopause was defined as natural menopause before the age of 45 years.

**Results:**

Mean- and menopausal age of women were reported as 59.3±7.94 and 48.6±4.81 years, respectively. No statistically significant linear association was observed between the daily intakes of lactose and galactose and the odds of early menopause. After adjusting for age, energy intake, and age at menarche, women in the middle tertiles of lactose (62%, 95%CI: 1.07, 2.46) and galactose (58%, 95% CI: 1.05, 2.39) intake had significantly higher odds of early menopause, than those in the first tertile. When the daily intake of lactose and galactose were expressed as the percentage of energy intake, the higher odds of early menopause among women in the middle tertile compared to those with the first tertile were reduced and became non-significant.

**Conclusion:**

No statistically significant linear associations were reported between the intake of lactose and galactose and age of menopause. However, the odds of early menopause in those women with the middle tertile of lactose and galactose intake were significantly higher than those women in the first tertile.

## Introduction

Menopause is an important physiological event for women, which can influence various aspects of their life. The estimated average age of natural menopause in developed and developing countries have been reported at 51 years and 48 years, respectively [1]. An early and late menopause can increase the incidence of health disorders and morbidities, including cardiovascular diseases and breast cancer in women [2]. Age at menarche, educational levels, parity, smoking, and BMI can influence the age at which menopause occurs [3–5].

It has been suggested that nutritional factors can also be associated with ovarian aging and menopausal age [6, 7]. An early depletion of ovarian reserves in rodent models with 35% galactose intake shows the possibility of an ovotoxicity effect of galactose [8, 9]. Reduction of ovarian reserves and early menopause in women with classic galactosemia also confirmed this toxic effect on ovaries [10]. A daily intake of ≥ 6 gr galactose in women without galactosemia is associated with a higher concentration of the follicle stimulating hormone (FSH), an indicator of menopause [11]. One of the possible mechanisms for ovotoxicity of galactose is the role of galactose in producing the advanced glycation end products (AGEs). Advanced glycation end products (AGEs) are certain heterogeneous compounds which are produced by reactions between reducing sugars and free amino groups of proteins, lipids or nucleic acids [12, 13]. As reducing sugars, monosaccharides (glucose, galactose, and fructose) have an open-chain aldehyde group [14], which can be oxidized via a redox reaction; AGEs are produced extensively by animal-derived foods and to a lesser extent by vegetables [15]. Several studies have shown that AGEs by retardation of ovarian follicles development can cause development of female reproductive aging [12, 16, 17]. However, the association between galactose consumption, ovarian reserve and age of menopause in women with the habitual amount of dietary galactose intake has not been studied, yet. Moreover, since only one study examined the association between lactose, the main source of dietary galactose, and age at menopause [18]; this study aimed at investigating the associations of the dietary lactose and galactose intakes with age at natural menopause.

## Materials and methods

### Design and sampling

**Fig 1:**
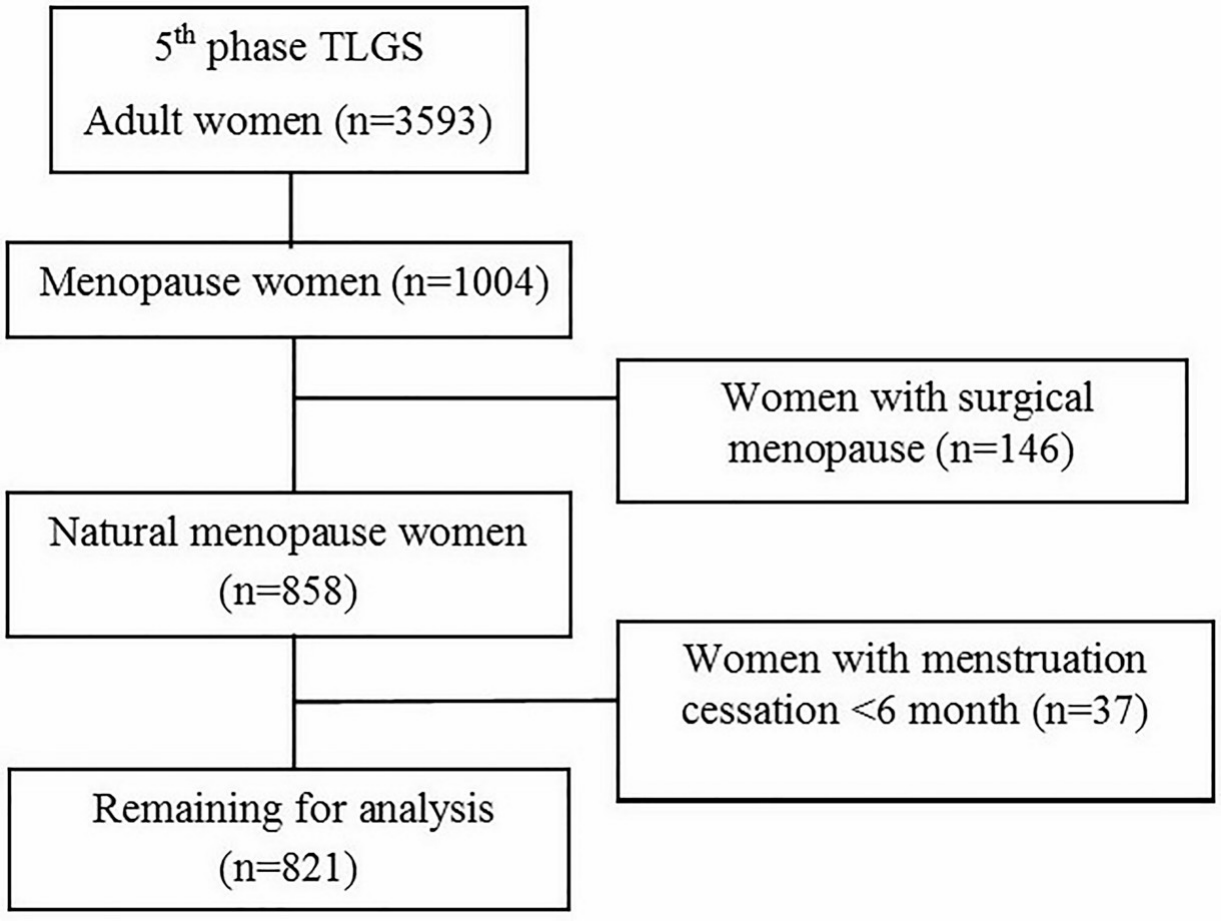
Selection of study participants.

The subjects of this cross-sectional study were recruited from among participants of the Tehran lipid and Glucose study (TLGS), an ongoing longitudinal study being conducted in Tehran, Iran. The aim and method of the study have been published elsewhere [19]. Briefly, the TLGS was initiated in 1999 with 15,005 participants, aged >3 years that were followed every three years. Data collected in the 5^th^ examination follow-up (2012–2015) was used in this study. Of 3593 adult women, who participated in the 5^th^ examination of the TLGS and had complete dietary data, 1004 reported menopause. After exclusion of women with surgical menopause (n = 146) and those with cessation of the menstrual period <12 months (n = 37), 821 women with natural menopause were included in this study. Details of the subjects’ selection are shown in (Fig 1). The ethics committee of the Research Institute for Endocrine Sciences (RIES) affiliated with Shahid Beheshti University of Medical Sciences approved the study research protocol, and written informed consent was obtained from all subjects.

### Non-dietary data

Socio-demographic and reproductive data, including age, age at menarche, education, smoking, the menstrual bleeding status, duration and cause of bleeding cessation, and marital status were determined using questionnaire. Menarche age was defined as the age at onset of menstruation for the first time. Menopause was defined as the cessation of menstrual bleeding for at least 12 months. Surgical menopause was defined as unilateral and bilateral oophorectomy. Early menopause was defined as the occurrence of natural menopause before the age of 45 years. Women who smoked daily or occasionally were categorized as smokers.

Weight was measured when the subjects were minimally clothed and without shoes, using a digital scale to the nearest 100 g. Height was measured in a standing position without wearing shoes using an upstretched measuring tape. The body mass index (BMI) was calculated as weight in kilograms divided by height in square meters.

### Dietary data

The habitual dietary intakes of participants during the past 12 month were assessed using a food-frequency questionnaire (FFQ) consisting of 168 items. The frequency consumption of each food item during the past year based on its portion size was asked from participants. Dietary intakes of nutrients were estimated by multiplying the consumption of each food item by nutrient contents, using the USDA Food Composition Table (FCT) [20].

Daily intakes of lactose was estimated from milk and dairy products including milk, yoghurt, cheese, ice-cream, and doogh (a traditional yoghurt drink); of these, milk had highest lactose content (5.20 g/100 g). Free galactose intakes was calculated from intakes of milk, yoghurt, cheese, some fruits (peaches, cherries, melons, fig, and kiwifruits), nuts (hazelnut and almond), and honey; of these honey has the highest galactose content (3.1g/100g).

The validity of the dietary questionnaire has been confirmed in a random sample of 132 participants from a cohort using 12 dietary recalls [21].

### Statistical analysis

Characteristics of participants, daily energy, and lactose and galactose intake were compared between the groups of women with early and non-early menopause using the student^’^s t-test and Mann-Whitney U tests for normal- and non-normal distributed quantitative variables, respectively; Chi-square test was used for categorical variables. Results were reported using mean ± standard deviation (SD), median (interquartile range), and number and percentage (%).

Participants were divided into three groups, based on dietary intakes of lactose and galactose. Logistic regression was used to determine the odds of early menopause in each tertile of lactose and galactose intake in non-adjusted model and after adjusting for potential covariates. In this study, age (continuous), education (university/non-university education), marital status (married/single), occupation (employed/unemployed), BMI (continuous), and smoking (smoker/non-smoker) were considered as potential covariates based on previous studies; of these variables, those significantly associated with odds of early menopause (p≤0.05) were included in the adjusted model. Energy intake was additionally included in the adjusted model to assess the association of lactose and galactose intakes with early menopause, independent of energy intakes. Results have been reported as the odds ratios (ORs) with a 95% confidence intervals (CIs). Statistical analyses were performed using the SPSS v.20 (IBM Corp., Chicago, Illinois, USA). P-values <0.05 were considered statistically significant.

## Results

The mean age and age at menopause in women were reported as 59.3±7.94 and 48.6±4.81 years, respectively. Of participants, 20.9% of the women experienced menopause before the age of 45 years. In comparison to women with menopause age >45 years, those with an early menopausal age were younger (p = 0.003), reached menarche earlier (p = 0.022), and had a higher intake of daily dietary energy (p = 0.048). Mean daily intakes of lactose and galactose did not differ significantly between the two groups (Table 1).

**Table 1:** Characteristics of participants based on age of menopause ^1^.

Variables	Early menopause ^2^ (n = 172)	Non-early menopause^3^ (n = 649)	p-value	Total participants
Age, years	57.4±9.4	59.7±7.4	0.003	59.3±7.9
Body mass index, kg/m^2^	30.8±3.4	30.4±4.8	0.325	30.4±5.1
BMI ≥25 kg/m^2^, n (%)	148 (86.0)	572 (88.1)	0.454	720 (87.7)
Smoker, n (%)	7(4.1)	18(2.7)	0.452	25(3)
Married, n (%)	124 (72.1)	486 (74.9)	0.493	610 (74.3)
Education (university graduates), n (%)	19 (12.4)	57 (9.5)	0.293	76 (10.5)
Employment, n (%)	13 (7.6)	28 (4.3)	0.112	41 (5.0)
Age at menopause, years	41.4±3.4	50.5±2.9	<0.001	48.6±4.8
Age at menarche, years	13.3±1.5	13.6±1.5	0.022	13.6±1.5
Total energy intake, Kcal/day	2142 (1720, 2720)	2011 (1574, 2586)	0.048	2046 (1592, 2605)
Lactose intake*, g/day	13.4 (7.03, 20.1)	12.9 (6.55, 20.9)	0.589	13.0 (6.59, 20.87)
*Galactose intake, g/day	2.6 (1.33, 3.88)	2.6 (1.16, 3.92)	0.835	2.6 (1.18, 3.91)

^1^Data are presented as mean±SDs, median (interquartile range), and n (%).

^2^ age of menopause ≤ 45 years

^3^ age of menopause > 45 years.

*Daily gram intakes of lactose and galactose were calculated using the frequency consumption of each food item and its nutrient contents via the USDA Food Composition Table (FCT).

The odds of early menopause based on the tertile of lactose and galactose intake are reported in Table 2.

**Table 2:** Odds ratios (ORs) for early menopause (age ≤45 years) based on daily intakes of lactose and galactose*.

	Tertiles of dietary intakes		
	Low	Middle	High
**Lactose (g/day)**			
Range	≤9.08	9.09–17.2	≥17.2
Early menopause (n)	48	71	53
Univariate	1	1.62(1.07, 2.46)	1.13(0.74, 1.75)
Adjusted^1^	1	1.56(1.03, 2.37)	1.05(0.66, 1.66)
**Lactose (% of energy)			
Range	≤0.4	0.4–0.8	≥0.8
Early menopause (n)	58	61	53
Univariate	1	1.02(0.68–1.54)	1.00(0.66–1.50)
Adjusted	1	1.04(0.68–1.58)	1.05(0.69–1.60)
**Galactose (g/day)**			
Range	≤1.5	1.5–3.6	≥3.6
Early menopause (n)	49	70	53
Univariate	1	1.58(1.05, 2.39)	1.08(0.70, 1.66)
Adjusted	1	1.56(1.02, 2.37)	1.01(0.67, 1.59)
**Galactose (% of energy)			
Range	≤0.08	0.09–0.1	≥0.1
Early menopause (n)	54	69	49
Univariate	1	1.18(0.78–1.78)	0.97(0.64–1.48)
Adjusted	1	1.19 (0.78–1.80)	1.01(0.66–1.55)

^1^ Adjusted for age, energy intake, and age at menarche.

* Daily gram intakes of lactose and galactose were calculated using the frequency consumption of each food item and its nutrient contents via the USDA Food Composition Table (FCT).

** Total intakes of lactose and galactose per 100 Kcal of food consumed.

No statistically significant linear association was observed between daily intakes of lactose or galactose and odds of early menopause. However, women in the middle tertiles of lactose (62%, 95%CI: 1.07, 2.46) and galactose (58%, 95% CI: 1.05, 2.39) intakes had significantly higher odds of early menopause, than those in the first tertile. These associations remained significant after adjustment of age, energy intake, and age at menarche. When daily intakes of lactose and galactose were expressed as percentage of energy intake, the higher odds of early menopause of women in middle tertile, compared to those in the first tertiles were reduced and became non-significant.

## Discussion

The association between intakes of habitual lactose and galactose with the timing of menopause was investigated in this study. No significant linear associations were observed between intakes of lactose or galactose, and menopause age. However, the odds of early menopause in those women with intakes of middle tertile of lactose (9.09–17.2 gr/day) and galactose (1.5–3.6 gr/day) were significantly higher than those women in the first tertile.

Decrease in follicular reserve and ovulatory response, increased atresia in ovarian follicles, and impaired migration of primordial germ cells (PGC) in rodent models with a high galactose diet, indicating the ovotoxic effects of galactose [8, 14]. A high frequent premature ovarian insufficiency (POI), infertility, ovarian dysfunction, and early menopause in women with galactosemia have also been attributed to the accumulation of galactose [9, 22]. It has also been shown that age of menopause in galactosemic women was associated with galactose-1-phosphat uridylyltransferase (GALT) enzyme deficiency, and age of menopause occurred earlier in women with the low enzyme activity [23]. In non-galactosemic women, a cross-sectional study suggested that the amount of galactose intakes but not galactose transferase activity may be associated with FSH concentrations, a marker of ovarian aging [11]; in this study, the serum levels of FSH in women with galactose intakes ≥ 6 g/day were significantly higher than in women with intakes <6 g/day. Whether or not the amount of galactose intake in the usual diet affect ovarian aging in non-galactosemic women, i.e., the timing of occurrence of menopause, is not yet clear. In contrast to the above mentioned studies suggesting the ovotoxicity effects of galactose, a prospective study showed that higher lactose intakes, the main dietary source of galactose, in premenopausal women aged <51 years could predict a later age of menopause [18].

According to our findings, there is no statistically significant linear associations between the intakes of lactose and galactose and age of menopause. However, the odds of early menopause in those women with the middle tertiles of lactose and galactose intake were significantly higher than those women in the first tertiles; a finding for which we have no justification.

It can be assumed that galactose affects ovarian function only of extremely high exposure (35% galactose) as seen in rodent models [8, 9]. In addition, sensitivity to galactose intake may differ based GALT enzyme activity, and therefore it can be hypothesized that women with total or partial activity deficit of the enzyme are more prone to the galactose intake than in women with normal enzyme activity. It is presumed that galactosemic women are more sensitive to dietary galactose, regardless of the amount of intakes mentioned earlier (<6 gr/day) than in those non-galactosemic counterparts. In addition, the effect of galactose on the ovaries seems to be dependent on time of initiation of exposure of embryo. Primary reserve follicles appear to be influenced by high galactose diet only during embryonic period [8, 9]. Exposure to high galactose after the embryonic stage can reduce ovulatory response, which is reversible after cessation of galactose intake [24].

The cross-sectional design is the main limitation of this study. Dietary intakes during the past 12 months were used to assess the long term habitual intakes in this study. However, menopausal transition may be accompanied by some changes in dietary intakes, which have not been considered in this study [25]. Information on menopausal status and age at menopause are self-reported, and therefore recall bias is likely to occur. Although this study was conducted among non-galactosemic women, the activity levels of enzymes involved in metabolism of lactose and galactose (lactase and galactose transferase) are important in response to lactose and galactose intakes, which were not measured in this study.

In conclusion, our data suggest that dietary lactose and galactose intakes are not associated with age of menopause in non-galactosemic women. Prospective studies investigating dietary intakes of lactose and galactose during premenopausal ages in women with different lactose and/ or galactose metabolizing enzymes activity levels are required to clarify the effects of galactose on ovarian aging.

## Supporting information

S1 Data Supporting data. (SAV)
